# Supplementary material for: A whole-animal phenotypic drug screen identifies suppressors of atherogenic lipoproteins
Source: eLife. 2026 Aug 27;14:RP105314. doi: 10.7554/eLife.105314 (PMC13521566; doi:10.7554/eLife.105314)
Supplement: Supplementary file 2 [file elife-105314-code1.zip › Output_data/JHDL_hit_summary_boxplot_2.pdf]

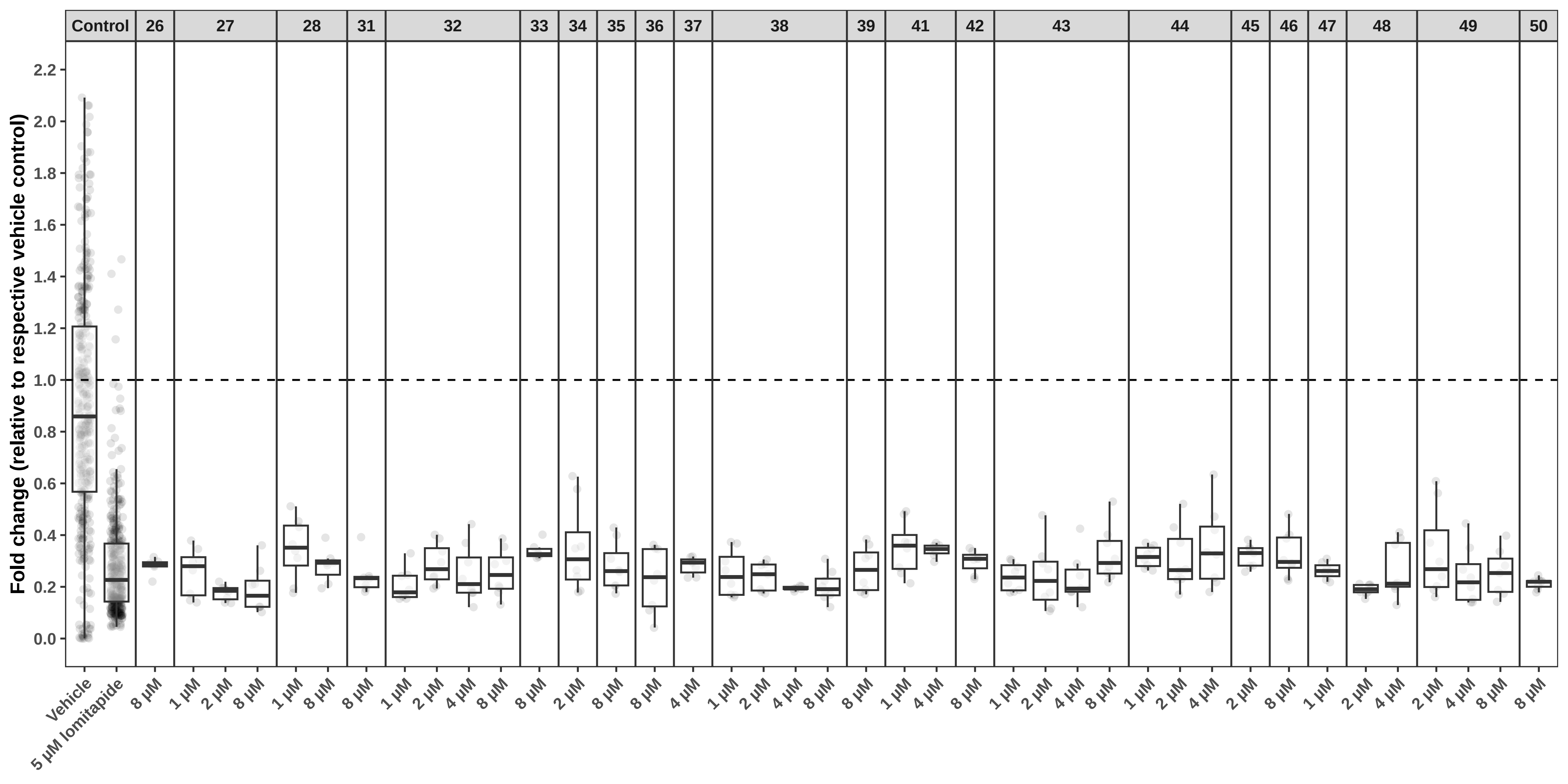
Control
26
27
28
31
32
33
34
35
36
37
38
39
41
42
43
44
45
46
47
48
49
50
Fold change (relative to respective vehicle control)
0.0
0.2
0.4
0.6
0.8
1.0
1.2
1.4
1.6
1.8
2.0
2.2
Vehicle
5 μM lomitapide
8 μM
1 μM
2 μM
8 μM
1 μM
8 μM
8 μM
1 μM
2 μM
4 μM
8 μM
8 μM
2 μM
8 μM
8 μM
4 μM
1 μM
2 μM
4 μM
8 μM
8 μM
1 μM
4 μM
8 μM
1 μM
2 μM
4 μM
8 μM
1 μM
2 μM
4 μM
2 μM
8 μM
1 μM
2 μM
4 μM
2 μM
4 μM
8 μM
8 μM
